# Reliability and validation of the Dutch Achilles tendon Total Rupture Score

**DOI:** 10.1007/s00167-016-4242-7

**Published:** 2016-07-14

**Authors:** K. T. M. Opdam, R. Zwiers, J. I. Wiegerinck, A. E. B. Kleipool, R. Haverlag, J. C. Goslings, C. N. van Dijk

**Affiliations:** 1 Department of Orthopaedic Surgery, Orthopaedic Research Center Amsterdam, Academic Medical Center, University of Amsterdam, Meibergdreef 9, 1105 AZ Amsterdam, The Netherlands; 2 Department of Orthopaedic Surgery, Onze Lieve Vrouwe Gasthuis, Oosterpark 9, 1091 AC Amsterdam, The Netherlands; 3 Department of Surgery, Onze Lieve Vrouwe Gasthuis, Oosterpark 9, 1091 AC Amsterdam, The Netherlands; 4 Trauma Unit, Academic Medical Center, University of Amsterdam, Meibergdreef 9, 1105 AZ Amsterdam, The Netherlands

**Keywords:** Achilles tendon rupture, ATRS, Cross-cultural, Reliability, Validity, PROM, Dutch

## Abstract

**Purpose:**

Patient-reported outcome measures (PROMs) have become a cornerstone for the evaluation of the effectiveness of treatment. The Achilles tendon Total Rupture Score (ATRS) is a PROM for outcome and assessment of an Achilles tendon rupture. The aim of this study was to translate the ATRS to Dutch and evaluate its reliability and validity in the Dutch population.

**Methods:**

A forward–backward translation procedure was performed according to the guidelines of cross-cultural adaptation process. The Dutch ATRS was evaluated for reliability and validity in patients treated for a total Achilles tendon rupture from 1 January 2012 to 31 December 2014 in one teaching hospital and one academic hospital. Reliability was assessed by the intraclass correlation coefficients (ICC), Cronbach’s alpha and minimal detectable change (MDC). We assessed construct validity by calculation of Spearman’s rho correlation coefficient with domains of the Foot and Ankle Outcome Score (FAOS), Victorian Institute of Sports Assessment-Achilles questionnaire (VISA-A) and Numeric Rating Scale (NRS) for pain in rest and during running.

**Results:**

The Dutch ATRS had a good test–retest reliability (ICC = 0.852) and a high internal consistency (Cronbach’s alpha = 0.96). MDC was 30.2 at individual level and 3.5 at group level. Construct validity was supported by 75 % of the hypothesized correlations. The Dutch ATRS had a strong correlation with NRS for pain during running (*r* = −0.746) and all the five subscales of the Dutch FAOS (*r* = 0.724–0.867). There was a moderate correlation with the VISA-A-NL (*r* = 0.691) and NRS for pain in rest (*r* = −0.580).

**Conclusion:**

The Dutch ATRS shows an adequate reliability and validity and can be used in the Dutch population for measuring the outcome of treatment of a total Achilles tendon rupture and for research purposes.

**Level of evidence:**

Diagnostic study, Level I.

**Electronic supplementary material:**

The online version of this article (doi:10.1007/s00167-016-4242-7) contains supplementary material, which is available to authorized users.

## Introduction

The Achilles tendon is the most frequently ruptured tendon in the human body with an increasing incidence from 4.7/100,000 in 1981 to 32.6/100,000 in 2002 [10]. Optimal treatment of Achilles tendon ruptures remains controversial. Despite extensive research, there is still no consensus about the best treatment option for Achilles tendon ruptures, namely operative or conservative treatment [6]. Patient-reported outcome measures (PROMs) are an addition in the evaluation of the treatment of patients in the clinical practice and contribute to research purposes [9].

To date, no Dutch PROM is validated for Achilles tendon ruptures specifically. For the Dutch population, several ankle-specific PROMs are available, such as the Foot and Ankle Outcome Score (FAOS) and Foot and Ankle Ability Measure (FAAM) [16, 17, 21]. There is one Dutch PROM for patients with Achilles tendinopathy specifically, the VISA-A-NL [20]. However, the VISA-A-NL is only developed for evaluation of symptoms and their effect on physical activity. The advantage of a disease-specific PROM is that it is more sensitive to change compared to a generic health-related quality of life instrument and can therefore measure the outcome after treatment more specific [14, 22].

In 2007, a patient-reported outcome measure specific for Achilles tendon ruptures was developed: the Achilles tendon Total Rupture Score (ATRS) [13]. The ATRS is validated in several languages, including English, Danish, Turkish and Persian [1, 2, 4, 8, 9].

As there is a lack of validated Dutch instruments for measuring outcome related to symptoms and physical activity after treatment of Achilles tendon ruptures specifically, there is a need for a validated translation of the ATRS for Dutch speaking individuals.

Therefore, the aim of this study was to translate the ATRS to a Dutch language version of the Achilles tendon total rupture score and to evaluate its measurement properties.

## Materials and methods

### Translation procedure

The validated English ATRS was translated into the Dutch language according to the guidelines of cross-cultural adaptation [5]. Forward translations were performed by two native English speakers who fluently spoke Dutch, and the backward translation was performed by two Dutch native speakers who fluently spoke English. A diverse group of ten volunteers checked for clarity of the wording and meaning of the questions. If there were discrepancies, this was dissolved with discussion.

### Reliability and validity evaluation

#### Patients

Patients treated for a total Achilles tendon rupture from 1 January 2012 to 31 December 2014 in the Academic Medical Center or Onze Lieve Vrouwe Gasthuis were recruited. The eligibility criteria were: age above 18 years, isolated unilateral Achilles tendon rupture without other serious lower limb injury and the ability to read, write and understand Dutch. Patients were invited by mail. To optimize the response rate, patients were given the choice to fill in the questionnaire sent to them by mail or by email. Two weeks after completing the first questionnaire, patients again received a questionnaire by mail or by email and were asked to complete the ATRS questionnaire second time. Only complete ATRS questionnaires were included in the analysis.

#### Outcome measures

All questionnaires contained the in Dutch translated version of the ATRS, a validated Dutch FAOS, VISA-A and Numeric Rating Scale for Pain in rest and during activity (NRS). For the second questionnaire, one anchor question was added, to determine whether the status of the Achilles tendon complaints had changed. We used the COnsensus-based Standards for the selection of health Measurement INstruments (COSMIN) checklist for validation of PROMs developed by the COSMIN Initiative [11, 12, 19].

The Achilles tendon Total rupture score (ATRS) is a patient-reported instrument and disease-specific tool designed to evaluate symptoms and physical activity in patients with Achilles tendon rupture. The ATRS is a self-administered instrument and contains ten items, each an 11-points Likert scale (0–10). A maximal score of 100 indicates no symptoms and full function, whereas a minimum score of 0 indicates severe symptoms and major limitations [13]. However, like the English version, the numbers were changed just to not make the patients confused, since they are used to ten being the worst, meaning that a minimum score of 0 indicates no symptoms and full function, whereas a maximum score of 100 indicates severe symptoms and major limitations.

The Foot and Ankle Outcome Score (FAOS) is a 42-items self-administered questionnaire originally designed to evaluate patients with ankle ligament injuries [15]. The FAOS has thus far been used in patients with lateral ankle instability, plantar fasciitis and Achilles tendon rupture, but was not specifically developed for Achilles tendon pathologies. The FAOS consists of five subscales: pain, other symptoms, activity in daily living (ADL), recreational and sport activities and foot and ankle-related quality of life (QOL). In 2014, the FAOS was validated for the Dutch language [16]. Each question in the FAOS is answered on a five-point Likert scale ranging from 0 to 4. A normalized score (100 indicating no symptoms and 0 indicating extreme symptoms) was calculated for each subscale. If there were one or two answers missing in the questionnaire, it was allowed to substitute the missing value by the mean value of the subscale [15].

The Victorian Institute of Sports Assessment-Achilles questionnaire (VISA-A-NL) is a PROM consisting of eight questions, validated for evaluation of pain, symptoms and their effect on physical activity specifically in patients with Achilles tendinopathy [20]. The score ranges from 0 to 100, where 0 represent the worst score and 100 the best score.

The Numeric Rating Scale for pain (NRS) is a common and practical method for assessing pain severity in rest and during activity such as running. In this study, a 11-point numeric rating scale was used, where patients are requested to quantify the intensity of their pain on a scale from 0 to 10 with 0 indicating no pain and 10 indicating the worst pain imaginable [7]. We assessed the NRS in rest and during running.

### Reliability

In this study, the reliability was assessed by the internal consistency, the reproducibility (test–retest) and the measurement error [12].

Internal consistency was defined as the degree of the interrelatedness among the items of the ATRS [12]. To measure interrelatedness among items, we used the Cronbach’s alpha with 0.7–0.95 assigned as good interrelatedness [18].

The test–retest reliability was defined as the ability of the Dutch ATRS to measure the same outcome twice in patients with an unchanged state of condition of the complaints [12]. Patients who reported a change in their state were excluded from the test–retest analysis. We assessed the test–retest reliability by calculation of the intraclass correlation coefficients (ICC-agreement, type 3 two-way mixed model) [3]. An ICC of 0 indicated there is no agreement between the two questionnaires, and an ICC of 1 means there was a perfect agreement. An ICC > 0.7 was considered as good agreement [23].

Measurement error was defined as the systematic and random error of a patient’s score that is not attributed to true changes in the construct of the ATRS [12]. Measurement error was calculated as the standard error of measurement (SEM) which was calculated as standard deviation (SD) × √(1 − reliability coefficient) [23]. From this SEM, the minimal detectable change (MDC) at individual level was calculated as 1.96 × √2 × SEM, and the MDC at group level was calculated by dividing the MDC at individual level by √*n* [23].

### Construct validity

Construct validity was defined as the degree to which the scores of the Dutch ATRS were consistent with the hypotheses stated below [12]. Due to the lack of a ‘golden standard’, the construct validity of the Dutch ATRS was assessed in terms of consistency to the subscales of the FAOS, VISA-A-NL and NRS in rest and during running [12]. The ATRS was compared with the FAOS subscales, VISA-A-NL and NRS in rest and during running by analysing means of Spearman’s correlation coefficients. Correlation coefficients between 0.4 and 0.7 (or between −0.4 and −0.7) were defined as a moderate correlation, coefficients lower than 0.4 or higher than −0.4 unconnected or measuring dissimilar constructs and coefficients above 0.7 or lower than −0.7 as strong correlation [18].

A priori hypotheses on correlation between the Dutch ATRS and the Dutch subscales of FAOS, VISA-A-NL and NRS were formulated to evaluate the construct validity. It was hypothesized that the Dutch ATRS would correlate strongly with the VISA-A-NL, the Dutch FAOS symptoms, FAOS function, FAOS pain, FAOS ADL and NRS during running, because the ATRS is a disease-specific tool designed to evaluate symptoms and physical activity and should measure similar construct. Therefore, it was hypothesized that the Dutch ATRS would correlate moderately with the FAOS QOL and NRS in rest since the QOL domain is not disease specific and the NRS in rest is not comparable to a status of activity. The construct validity was defined sufficient if at least 75 % of the results were in correspondence with these hypotheses [18].

### Interpretability

Interpretability was defined as the degree to which qualitative meaning can be assigned to the Dutch ATRS quantitative scores or changes in scores [12]. Interpretability was assessed by the distribution and occurrence of ceiling and floor effects. Floor or ceiling effects were considered to be present if more than 15 % of respondents achieved the lowest or highest possible score.

### Statistical analysis

Variables with a normal distribution were presented as the mean and standard deviation. Variables with a non-normal distribution were presented as the median and interquartile range, and the Kolmogorov–Smirnov test was used for data distribution assessment. Clinimetric properties were calculated as described above. All statistical analyses were performed with Statistical Package for Social Sciences (SPSS) version 22.0 (SPSS Inc. Chicago, IL).

## Results

During both of the forward and the backward translation, there were no discrepancies in translation to discuss and no adjustments were necessary. Ten volunteers checked the Dutch version of the questionnaire and found the questionnaire to be clear. Appendix 1 of ESM presents the Dutch ATRS questionnaire.

**Fig. 1:**
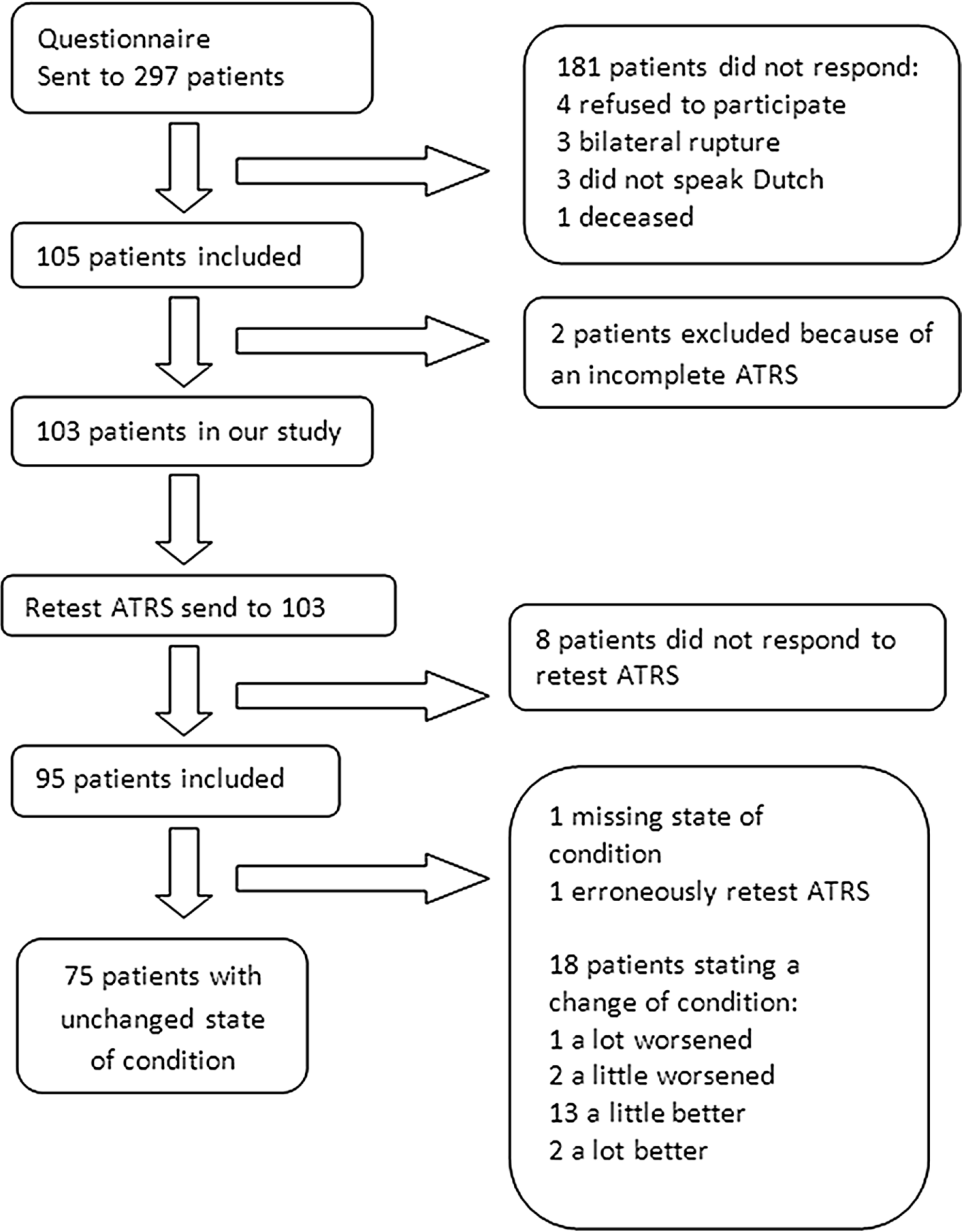
Inclusion flowchart

Questionnaires were sent to 297 patients who were treated for a total Achilles tendon rupture. A total of 105 (35 % response rate) patients filled out the questionnaires for the first time. Of these 105 questionnaires, 103 (98 %) were complete. Table 1 shows the characteristics of participants within the study and non-responders. The median time between injury and participation in this study was 20 months (IQR 13–29). Hereafter, 95 (92 %) patients completed the ATRS second time for the test–retest reliability (see Fig. 1). Test–retest reliability was calculated over 75 patients, since 18 patients reported a change in state of complaints, and in one questionnaire, the anchor question was missing and one patient did not fill out the retest ATRS completely. The median time between the questionnaires was 17 days (IQR 15–26).

**Table 1:** Characteristics of present study

	Non-responders (*N* = 181)	ATRS (*N* = 103)	Retest ATRS (*N* = 75)
	*N* (%)	*N* (%)	*N* (%)
Sex			
Male	139 (76.8)	76 (73.8)	56 (74.7)
Female	42 (23.2)	27 (26.2)	19 (25.3)
Age^a^			
Years	44.3 (SD 13.2)	50.2 (SD 13.6)	51.29 (SD 13.9)
Ankle			
Left	79 (43.6)	50 (48.5)	35 (46.7)
Right	102 (56.4)	53 (51.5)	40 (53.3)
Treatment			
Conservative	103 (56.9)	53 (51.5)	41 (54.7)
Operative	78 (43.1)	50 (48.5)	34 (45.3)
Time since rupture to questionnaire^b^			
In months	16.0 (IQR 8.0–25.0)	20.0 (IQR 13.0–29.0)	21.0 (IQR 14.0–29.0)
Athlete			
Yes		77 (74.8)	56 (74.7)
No		26 (25.2)	19 (25.3)
Questionnaire			
By mail		96 (93.2)	33 (44)
By email		7 (6.8)	42 (56)
Time between questionnaires^b^			
In days			17 (IQR 15–26)

^a^Data in mean (SD)

^b^Data in median (interquartile range)

### Missing data

At baseline, four values (0.4 %) of the 1050 (10 questions × 105 patients) ATRS questions were missing.

### Reliability

The Cronbach’s alpha of the Dutch ATRS was 0.96. The ICC of the test–retest reliability was 0.852. The SEM was 10.91, and the MDC was 30.2 at individual level and 3.5 at group level.

### Construct validity

Of the prior hypothesized correlations, 75 % was confirmed which indicates a sufficient construct validity. Table 2 presents the correlation coefficients assessed with Spearman’s rho between the Dutch ATRS and the Dutch subscales of the FAOS, VISA-A and NRS for pain.

**Table 2:** Construct validity measured by correlation coefficients

	ATRS	Hypothesis	Result
FAOS symptoms	0.77	Strong correlation	Strong correlation
FAOS pain	0.72	Strong correlation	Strong correlation
FAOS ADL	0.87	Strong correlation	Strong correlation
FAOS function	0.84	Strong correlation	Strong correlation
FAOS QOL	0.86	Moderate correlation	Strong correlation
VISA-A-NL	0.69	Strong correlation	Moderate correlation
NRS rest	−0.58	Moderate correlation	Moderate correlation
NRS running	−0.75	Strong correlation	Strong correlation

### Interpretability

No floor effect was identified, and none of the patients achieved the minimum score. Eight of the 103 patients (7.8 %) achieved the maximum score.

## Discussion

The most important finding of this study was that the Dutch ATRS shows an adequate reliability and validity and can be used in the Dutch population.

The internal consistency of the Dutch ATRS is high (0.96), and the test–retest reliability is good (ICC value of 0.852).

The high internal consistency is in agreement with the previous reported internal consistencies of the ATRS, ranging from 0.89 to 0.96 [4, 8, 9, 13]. Comparison of the test–retest reliability to previously reported data (ranging from 0.908 to 0.986), the ICC in this study was lower. This can be explained by the differences in time interval, ranging from 15 min to 21 days of the test–retest assessment in the previous studies [2, 4, 8, 13]. We aimed at a time interval of 14 days for the test–retest assessment since the ATRS is a questionnaire consisting of only ten items, and we wanted to prevent that patients remembered the answers of the first questionnaire. Possibly, the reliability estimates are understated because it is likely that we influenced the ICC due to the fact that 47 patients filled out the first assessment on paper and the second assessment online by which we compromised the reliability assessment. For reliability assessment, it is necessary to fill out the questionnaire twice under the same circumstances, and by using the different options of filling out the questionnaire, we could have influenced the test–retest reliability in a negative way.

The SEM in this study was higher in comparison with previous ATRS studies (3.2–6.673). As a result, the MDC on individual level (30.24) is large. This would mean that the questionnaire is not suitable for comparing individual patients since there is a difference needed of minimal 31 points to detect real change. However, the MDC at group level is 3.49 points which makes the ATRS suited for group evaluation. Of the other ATRS validation studies, only Carmont et al. [2] reported an MDC for comparison between groups of 6.75 points.

In previous validation studies of the ATRS, the correlation with many different PROMs (FAOS subscales [8, 13], VISA-A [4, 13], DRI [9], SF-12 [8], SF-36 [4] and EQ-5D [9]) has been determined. We correlated the ATRS with the FAOS, VISA-A and NRS, because those are validated in Dutch and are the most disease-specific PROMS available. Construct validity is supported by six out of eight of the hypothesized correlations (75 %). The correlation coefficients between the Dutch FAOS subscales and the Dutch ATRS were all strong, even though we expected that the QOL subscale would correlate moderate. Nilsson et al. [13] reported strong correlation with three out of five FAOS subscales (symptoms, sport and QOL) and moderate correlations for the pain and ADL. Kaya et al. [8] also had a strong correlation with three out of five subscales of the FAOS (pain, ADL and sport) and the subscales of symptoms and QOL correlated moderate. It was hypothesized that the Dutch ATRS would have a strong correlation with the VISA-A as well as the Danish and Swedish cohort, but it showed to have a moderate correlation.

None of the patients achieved the minimum score, and therefore, no floor effect was present which is comparable to the percentages of other studies, which vary from 0 to 1 % [4, 9]. Only 7.8 % achieved the maximum score which is below the threshold of 15 %, and in other studies, this percentage ranges from 0 to 14 % [4, 8, 9].

The number of missing items was low (0.4 %), and this can indicate that the questions are clear and are of value to the patient, since questions in questionnaires are often marked as not applicable by the patient or the online assessment could have been of influence.

A problem with validation of PROMS in general is a lack of a gold standard, and therefore, validity should be interpreted with caution. Although efforts were made to include as many participants as possible, only 105 out of 297 responded to our questionnaire. However, non-responders characteristics were not much different compared to the responders, and nevertheless, we were able to test the reliability. Furthermore, the time since rupture differed from 1 to 43 months after treatment which is a wide spread in time after injury and probably affects the results by making the group more heterogonous, because in daily practice evaluation only takes place after short time after rupture. However, the Dutch ATRS can be used in the Dutch population for group evaluation of treatment of a total Achilles tendon rupture and for research purposes. A next step for future studies is to assess responsiveness and to determine the minimum clinically important differences.

## Conclusion

In conclusion, the Dutch ATRS shows an adequate reliability and validity and can be used in the Dutch population for group evaluation of treatment of a total Achilles tendon rupture and for research purposes.

## Electronic supplementary material

Below is the link to the electronic supplementary material.

Supplementary material 1 (PDF 102 kb)
