# Supplementary material for: Reliability and validation of the Dutch Achilles tendon Total Rupture Score
Source: Knee Surg Sports Traumatol Arthrosc. 2016 Jul 14;26(3):862–8. doi: 10.1007/s00167-016-4242-7 (PMC5847201; doi:10.1007/s00167-016-4242-7)
Supplement: Supplementary file 1 — Supplementary material 1 (PDF 102 kb) [file 167_2016_4242_MOESM1_ESM.pdf]

Appendix I Dutch ATRS

Achillespees Ruptuur Score (ATRS)

Alle vragen hebben betrekking op uw beperkingen/moeilijkheden met betrekking tot uw aangedane achillespees.
Plaats een kruisje in het hokje dat het beste bij uw niveau van beperking past.

De schaal loopt van 0 (geen problemen) tot 10 (onmogelijk).

1. **Wordt u beperkt door een verminderde kracht in de kuit/achillespees/voet?**

☐ 0	☐ 1	☐ 2	☐ 3	☐ 4	☐ 5	☐ 6	☐ 7	☐ 8	☐ 9	☐ 10
Geen Probleem										Onmogelijk

2. **Wordt u beperkt door vermoeidheid/een vermoeid gevoel in de kuit/achillespees/voet?**

☐ 0	☐ 1	☐ 2	☐ 3	☐ 4	☐ 5	☐ 6	☐ 7	☐ 8	☐ 9	☐ 10
Geen Probleem										Onmogelijk

3. **Wordt u beperkt door stijfheid/een stijf gevoel in de kuit/achillespees/voet?**

☐ 0	☐ 1	☐ 2	☐ 3	☐ 4	☐ 5	☐ 6	☐ 7	☐ 8	☐ 9	☐ 10
Geen Probleem										Onmogelijk

4. **Wordt u beperkt door pijn in de kuit/achillespees/voet?**

☐ 0	☐ 1	☐ 2	☐ 3	☐ 4	☐ 5	☐ 6	☐ 7	☐ 8	☐ 9	☐ 10
Geen Probleem										Onmogelijk

5. **Wordt u beperkt tijdens activiteiten in het dagelijks leven?**

☐ 0	☐ 1	☐ 2	☐ 3	☐ 4	☐ 5	☐ 6	☐ 7	☐ 8	☐ 9	☐ 10
Geen Probleem										Onmogelijk

6. **Wordt u beperkt/ ondervindt u moeilijkheden bij het lopen op oneffen ondergrond.**

☐ 0	☐ 1	☐ 2	☐ 3	☐ 4	☐ 5	☐ 6	☐ 7	☐ 8	☐ 9	☐ 10
Geen Probleem										Onmogelijk

7. **Wordt u beperkt wanneer u snel een trap of heuvel op loopt?**

☐ 0	☐ 1	☐ 2	☐ 3	☐ 4	☐ 5	☐ 6	☐ 7	☐ 8	☐ 9	☐ 10
Geen Probleem										Onmogelijk

8. **Wordt u beperkt tijdens activiteiten waarbij u moet rennen?**

☐ 0	☐ 1	☐ 2	☐ 3	☐ 4	☐ 5	☐ 6	☐ 7	☐ 8	☐ 9	☐ 10
Geen Probleem										Onmogelijk

9. **Wordt u beperkt tijdens activiteiten waarbij u moet springen?**

☐ 0	☐ 1	☐ 2	☐ 3	☐ 4	☐ 5	☐ 6	☐ 7	☐ 8	☐ 9	☐ 10
Geen Probleem										Onmogelijk

10. **Wordt u beperkt bij het verrichten van zwaar lichamelijk werk?**

☐ 0	☐ 1	☐ 2	☐ 3	☐ 4	☐ 5	☐ 6	☐ 7	☐ 8	☐ 9	☐ 10
Geen Probleem										Onmogelijk

Totaalscore:______
